# Multiparametric Integrated ^18^F-FDG PET/MRI-Based Radiomics for Breast Cancer Phenotyping and Tumor Decoding

**DOI:** 10.3390/cancers13122928

**Published:** 2021-06-11

**Authors:** Lale Umutlu, Julian Kirchner, Nils Martin Bruckmann, Janna Morawitz, Gerald Antoch, Marc Ingenwerth, Ann-Kathrin Bittner, Oliver Hoffmann, Johannes Haubold, Johannes Grueneisen, Harald H. Quick, Christoph Rischpler, Ken Herrmann, Peter Gibbs, Katja Pinker-Domenig

**Affiliations:** 1 Department of Diagnostic and Interventional Radiology and Neuroradiology, University Hospital Essen, University of Duisburg-Essen, D-45147 Essen, Germany; Johannes.Haubold@uk-essen.de (J.H.); Johannes.grueneisen@uk-essen.de (J.G.); 2 Department of Radiology, Memorial Sloan Kettering Cancer Center, New York, NY 10065, USA; gibbsp@mskcc.org (P.G.); pinkerdk@mskcc.org (K.P.-D.); 3 Department of Diagnostic and Interventional Radiology, University Dusseldorf, Medical Faculty, D-40225 Dusseldorf, Germany; Julian.Kirchner@med.uni-duesseldorf.de (J.K.); Nils-Martin.Bruckmann@med.uni-duesseldorf.de (N.M.B.); Janna.Morawitz@med.uni-duesseldorf.de (J.M.); antoch@med.uni-duesseldorf.de (G.A.); 4 Institute of Pathology, University Hospital Essen, West German Cancer Center, University Duisburg-Essen and the German Cancer Consortium (DKTK) Essen, D-45147 Essen, Germany; Marc.Ingenwerth@uk-essen.de; 5 Department Gynecology and Obstetrics, University Hospital Essen, University of Duisburg-Essen, D-45147 Essen, Germany; ann-kathrin.bittner@uk-essen.de (A.-K.B.); Oliver.Hoffmann@uk-essen.de (O.H.); 6 Erwin L. Hahn Institute for Magnetic Resonance Imaging, University of Duisburg-Essen, D-45141 Essen, Germany; Harald.Quick@uk-essen.de; 7 High-Field and Hybrid MR Imaging, University Hospital Essen, University of Duisburg-Essen, D-45147 Essen, Germany; 8 Department of Nuclear Medicine, University Hospital Essen, University of Duisburg-Essen, D-45147 Essen, Germany; christoph.rischpler@uk-essen.de (C.R.); ken.herrmann@uk-essen.de (K.H.)

**Keywords:** multiparametric ^18^F-FDG PET/MRI, radiomics, breast cancer, radiomics-based phenotyping and tumor decoding

## Abstract

**Simple Summary:**

Breast cancer is considered the leading cancer type and main cause of cancer death in women. In this study, we assess simultaneous ^18^F-FDG PET/MRI of the breast as a platform for comprehensive radiomics analysis for breast cancer subtype. The radiomics-based analysis comprised prediction of molecular subtype, hormone receptor status, proliferation rate and lymphonodular and distant metastatic spread. Our results demonstrated high accuracy for multiparametric MRI alone as well as ^18^F-FDG PET/MRI as an imaging platform for high-quality non-invasive tissue characterization.

**Abstract:**

Background: This study investigated the performance of simultaneous ^18^F-FDG PET/MRI of the breast as a platform for comprehensive radiomics analysis for breast cancer subtype analysis, hormone receptor status, proliferation rate and lymphonodular and distant metastatic spread. Methods: One hundred and twenty-four patients underwent simultaneous ^18^F-FDG PET/MRI. Breast tumors were segmented and radiomic features were extracted utilizing CERR software following the IBSI guidelines. LASSO regression was employed to select the most important radiomics features prior to model development. Five-fold cross validation was then utilized alongside support vector machines, resulting in predictive models for various combinations of imaging data series. Results: The highest AUC and accuracy for differentiation between luminal A and B was achieved by all MR sequences (AUC 0.98; accuracy 97.3). The best results in AUC for prediction of hormone receptor status and proliferation rate were found based on all MR and PET data (ER AUC 0.87, PR AUC 0.88, Ki-67 AUC 0.997). PET provided the best determination of grading (AUC 0.71), while all MR and PET analyses yielded the best results for lymphonodular and distant metastatic spread (0.81 and 0.99, respectively). Conclusion: ^18^F-FDG PET/MRI enables comprehensive high-quality radiomics analysis for breast cancer phenotyping and tumor decoding, utilizing the perks of simultaneously acquired morphologic, functional and metabolic data.

## 1. Introduction

Breast cancer is considered the leading cancer type and main cause of cancer death in women. With increasing prevalence due to early diagnosis, alterations in risk factors and an aging population, early detection and early prediction of prognosis are two key factors for appropriate patient management [1]. Continuous progress in the understanding of proteogenomics and its relation to cancer has transitioned into a deeper appreciation for the importance of tumor decoding and phenotyping toward precision medicine. The introduction of radiomics as the conversion of the information and features contained in medical images into quantifiable data and the subsequent mining of these data has facilitated a new platform for imaging-based, non-invasive tissue characterization [2,3,4]. Radiomics is based on the hypothesis that the extracted imaging features correlate to genotypic and phenotypic characteristics of the breast tumor tissue [5,6]. Hence, it is considered a valuable new tool in the concept of personalized medicine [7]. 

Over the past 5 years, numerous trials have been performed to assess the value and validity of radiomics in breast cancer characterization [8,9,10]. As recently reported in a rapid review on radiomics and breast cancer, most studies put the focus on breast MRI with the main emphasis on morphology, contrast enhancement kinetics and restricted diffusivity as features. While functional features derived from MRI and ^18^F-FDG PET/CT have been shown to be valuable in the detection and characterization of breast cancer and lymph node involvement, only a very limited number of studies investigated the potential of integrated ^18^F-FDG PET/MRI for radiomics applications in breast cancer [11,12,13,14]. Hence, the aim of this study is to assess simultaneous ^18^F-FDG PET/MRI of the breast as a platform for comprehensive radiomics analysis for breast cancer subtype analysis, hormone receptor status, proliferation rate and lymphonodular and distant metastatic spread. 

## 2. Material and Methods

### 2.1. Patients 

This retrospective study was approved by the local ethics committee and patient written consent was waived due to the utilization of anonymized data. A total of 124 female patients with newly diagnosed, therapy-naïve breast cancer were included. All patients met the following inclusion criteria: (1) newly diagnosed, biopsy-proven, treatment- naïve, hormone receptor-positive (HR+) and/or human epidermal growth factor receptor-2 overexpression (Her2+) T2 tumor or higher T-stage or (2) newly diagnosed, treatment-naïve, HR/Her2-negative, i.e., triple-negative (TN) tumor, of any size or (3) newly diagnosed, treatment-naïve tumor with high molecular risk (T1c, Ki-67 > 14%, HER2 overexpression, G3). Exclusion criteria were former malignancies in the last 5 years, contraindications to MRI or MRI contrast agents and pregnancy or breast-feeding. Inclusion criteria were chosen to set elevated pre-test probability for distant metastases. 

### 2.2. PET/MRI

All ^18^F-FDG PET/MRI examinations were performed on an integrated 3-Tesla PET/MRI system (Biograph mMR, Siemens Healthcare GmbH, Erlangen, Germany) and obtained one hour after injection of a bodyweight-adapted dosage of ^18^F-FDG (4 MBq/kg bodyweight). The protocol included a dedicated breast ^18^F-FDG PET/MR and a whole-body imaging scan [15]. For this dedicated study, only the PET/MR breast examinations were considered. These were performed in head-first prone position utilizing a dedicated 16-channel breast radiofrequency (RF) coil (Rapid Biomedical, Rimpar, Germany) which was specifically developed and designed for use in integrated whole-body PET/MR imaging [16]. PET acquisition was performed simultaneously with MRI data acquisition in prone positioning with an acquisition time of 20 min/bed position. PET image reconstruction was performed subsequently, utilizing an iterative ordered-subset expectation maximization algorithm, 3 iterations and 21 subsets, a Gaussian filter with 4 mm full width at half maximum and a 256 × 256 image matrix for the breast and a 344 × 344 image matrix for the whole body. PET data were automatically attenuation corrected using the implemented 4-compartment model attenuation map (μ-map) calculated from fat-only and water-only datasets, as obtained by Dixon-based sequences.

**Figure 1:**
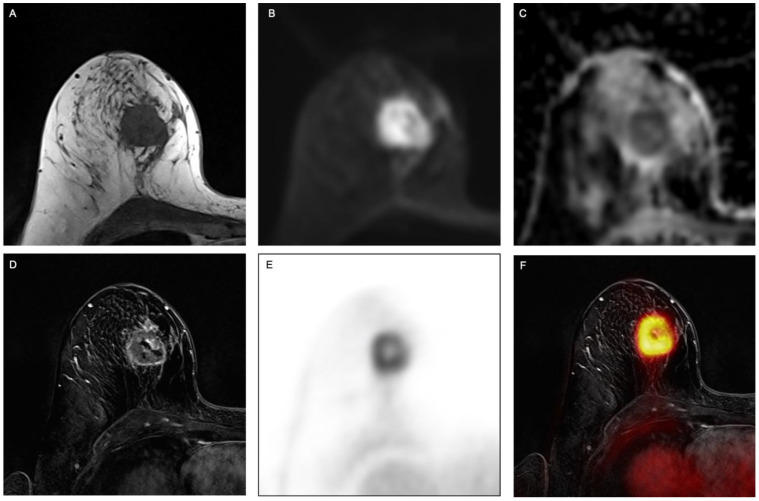
Example of a 63-year-old woman with invasive breast cancer in the right breast, clearly visible on (**A**) fat-saturated T2-weighted turbo spin-echo (TSE) sequence, (**B**) transversal diffusion-weighted echo-planar imaging (EPI) sequence with (**C**) apparent diffusion coefficient (ADC) as well as on (**D**) contrast-enhanced T1w images, (**E**) PET and (**F**) fused PET/MR images.

The dedicated breast protocol comprised the following sequences: (1) a transversal fat-saturated T2-weighted turbo spin-echo (TSE) sequence (2) a transversal diffusion-weighted echo-planar imaging (EPI) sequence with apparent diffusion coefficient (ADC) mapping, (3) six repetitions of a transversal 3-dimensional fast low-angle shot T1w (FLASH) sequence for dynamic contrast-enhanced imaging. A dose of 2ml/kg bodyweight gadoterate meglumine (Guerbet, Dotarem) was injected intravenously after the first FLASH sequence with a flow of 20 mL/s using an automated injector (Spectris Solaris, MR Injection System; Medrad, Pittsburg, PA). Subsequent automated image subtraction was performed. Please see Figure 1 for an example.

### 2.3. Image Analysis

Two board-certified radiologists with 13 and 5 years of experience in breast imaging and hybrid imaging and a nuclear medicine physician with 15 years of experience evaluated the ^18^F-FDG PET/MRI data. All images were imported into an open-source medical image viewer (Horos v. 3.3.5, LGPL, Annapolis, MD, USA) for image visualization and quantitative parameter extraction. Breast lesions were identified on DCE post-contrast subtracted images and lesion location was recorded.

### 2.4. Radiomics Analysis

PET/MRI images were imported to dedicated software (ITK-SNAP v. 3.6.0) [17] for lesion segmentation. A radiologist with 13 years of experience in breast imaging annotated each lesion on the subtracted second post-contrast time point using a semi-automated method. Inclusion of cystic/necrotic areas and/or biopsy markers was avoided during segmentation.

One hundred and one radiomic features were calculated for each patient with CERR software [18]. Features were calculated in six classes (22 first order, 26 based on gray-level cooccurrence matrices, 16 based on run length matrices, 16 based on size zone matrices, 16 based on neighborhood gray-level dependence matrices and 5 based on neighborhood gray tone difference matrices). CERR has recently been demonstrated to conform to the Image Biomarker Standardization Initiative (IBSI) guidelines. All images were reduced to 32 gray levels prior to radiomics feature calculations. All dynamic images were normalized to the pre-contrast phase, resulting in maps of percentage enhancement. To account for class imbalances present in the data, adaptive synthetic sampling was employed to equalize class sizes [19]. This prevented subsequent models from potentially classifying all cases as belonging to the majority class. 

### 2.5. Reference Standard 

Tumor histology, tumor and nuclear grade and immunohistochemical status, including estrogen receptor, progesterone receptor and HER2, were derived from either final histopathological results from surgical tumor specimens or, in the case of neoadjuvant chemotherapy, from pre-treatment image-guided biopsy. All diagnoses were made by a surgical pathologist specialized in breast cancer according to the 4th edition of the WHO classification of tumors. Tumor grade was determined according to Elston and Ellis [17]. For immunohistochemistry (IHC), 1.5 µm thick slides were incubated with antibodies against Ki-67 (Ventana, clone 30-9, ready to use), ER (Ventana, clone SP1, ready to use), PR (Ventana, clone 1E2, ready to use) or HER2 (Ventana, clone 4B5, ready to use). IHC was performed with an OptiView Ventana System (Ki-67, ER) or UltraView Ventana System (PR, HER2) according to the manufacturer’s protocol. Subsequent FISH analyses for ERBB2 (Her2) amplification were performed for tumors scored as 2+. For precise application of the FISH probe, the tumor areas of 1.5 µm sections were incubated with a ZytoLight SPEC ERBB2/CEN17 Dual Color Probe (ZytoVision). Signal enumeration was performed with a microscope (Leica DM6 B, Leica Microsystems CMS GmbH) and results classified according to ASCO/CAP Guidelines 2018 [20].

Proliferation index Ki-67 was recorded as <15% (low proliferation) or ≥15% (high proliferation) [21]. In the case of equivocal HER2 status, lesions were additionally evaluated using fluorescence in situ hybridization and classified as positive when gene amplification was detected.

Using the dichotomized immunohistochemical evaluation of these three receptors to derive molecular subtypes [22], breast tumors were classified into luminal A (ER/PR+/HER2−, Ki-67 < 15%), luminal B (ER/PR+/HER2−, Ki-67 > 15%), HER2+ (ER/PR+, HER2+ and ER/PR−, HER2+) and triple negative (ER/PR−, HER2−) [23,24].

For N-stage, histopathological samples for lymph node evaluation were present for all patients (patient-based analysis). In case neoadjuvant chemotherapy was administered before lymphadenectomy, additional histopathological preparations were evaluated, using focal fibrosis or focal necrosis as a retrospective indicator for previously vital lymph node metastases [25,26]. For M-stage, metastases were proven by CT (*n* = 1) or by CT-guided/surgical biopsy (*n* = 6). All of the non-histopathologically proven lesions were re-evaluated with all follow-up imaging and/or clinical follow-up to exclude malignancy.

### 2.6. Statistical Analysis and Predictive Model Building

LASSO regression was utilized to determine which radiomic features were of most importance. LASSO was employed due to its fast nature, its ability to avoid overfitting and the fact that it can be applied even when the number of features is greater than the number of cases/samples [27,28]. A maximum of 6 features were selected for each model to avoid overfitting. If fewer features were determined to be of importance, only those were forwarded for use in model development. Predictive models were then developed in Matlab using support vector machines and 5-fold cross validation. With insufficient data to perform training, validation and testing on distinct datasets, the choice of machine learning was a pragmatic one. A support vector machine was utilized since they are known to work well for small datasets, are memory efficient and usually provide good performance [29,30]. Models were developed utilizing the different data types in isolation (ADC, T2, PET, dynamic phase 1, dynamic phase 2, dynamic phase 3, dynamic phase 4, dynamic phase 5) and then in various combinations (all dynamic phases aggregated, all MR data aggregated, all imaging data aggregated). Diagnostic metric sensitivity, specificity, positive predictive value, negative predictive value and accuracy were calculated for each model.

## 3. Results

### 3.1. Patient Population and Breast Lesion Characteristics

The mean age of the 124 patients was 54 years (range 31–86 y). Fifty-five patients were pre-menopausal, 12 peri-menopausal and 57 post-menopausal. Malignant lesions comprised 109 invasive ductal cancers, no special type (NST), 7 invasive lobular carcinomas (ILC) and 8 other types. Of the 125 treatment-naïve, biopsy-proven breast cancers, 92 were ER+ (74%), 88 were PR+ (71%), 21 were HER2+ (17%), 111 were high proliferation with Ki-67 greater than 15% (90%). Seventeen cancers were classified as luminal A (14%), eighty-two as luminal B (66%), five as HER2-enriched (4%) and nineteen as TN (16%). Five cancers were classified as G1 (4%), 67 as G2 (54%) and 52 as G3 (42%). A total of 49 patients showed lymph node metastases (40%) and a total of seven patients distant metastases (6%). Please see Table 1 for all patient details. A selection of the best results achieved for each assessed parameter is shown in Table 2. For detailed results of radiomic-based classifications for every prediction, including detailed numbers of dedicated sequences (T2, ADC, dynamic 1-5, all dynamics (DCE), all MR, PET and all MR + PET), see Online Supplements Tables S1–S9 for diagnostic metrics and Tables S10–S18 for radiomics features utilized in the models.

### 3.2. Radiomics Analysis to Predict Subtype

**Figure 2:**
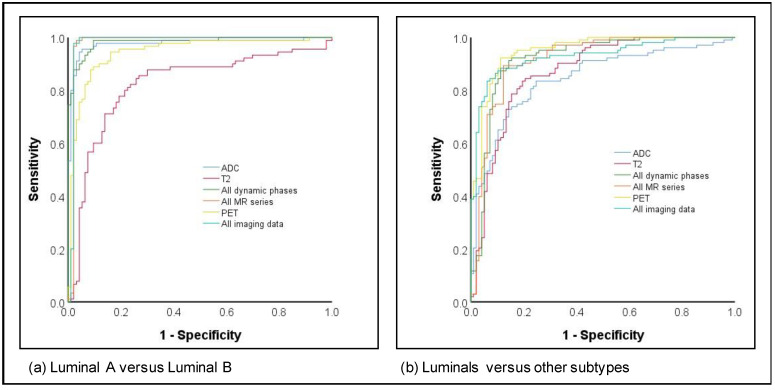
ROC curves for subtype prediction for (**a**) Luminal A versus Luminal B and (**b**) Luminals versus other subtypes.

The differentiation of luminal A versus luminal B cancers as well as luminals versus other subtypes both achieved high AUCs, with 11/12 image parameters yielding AUCs > 0.90 for luminal A versus luminal B and 5/12 for luminals versus others (Figure 2). While the highest AUC and accuracy for differentiation between luminal A and B were achieved by all MR sequences (AUC 0.98; accuracy 97.3%), the highest values for differentiation between luminals and other subtypes were achieved by PET imaging only (AUC 0.95; accuracy 88.5%).

### 3.3. Radiomics Analysis to Predict the Hormone Receptor Status, HER2 and Proliferation Rate

**Figure 3:**
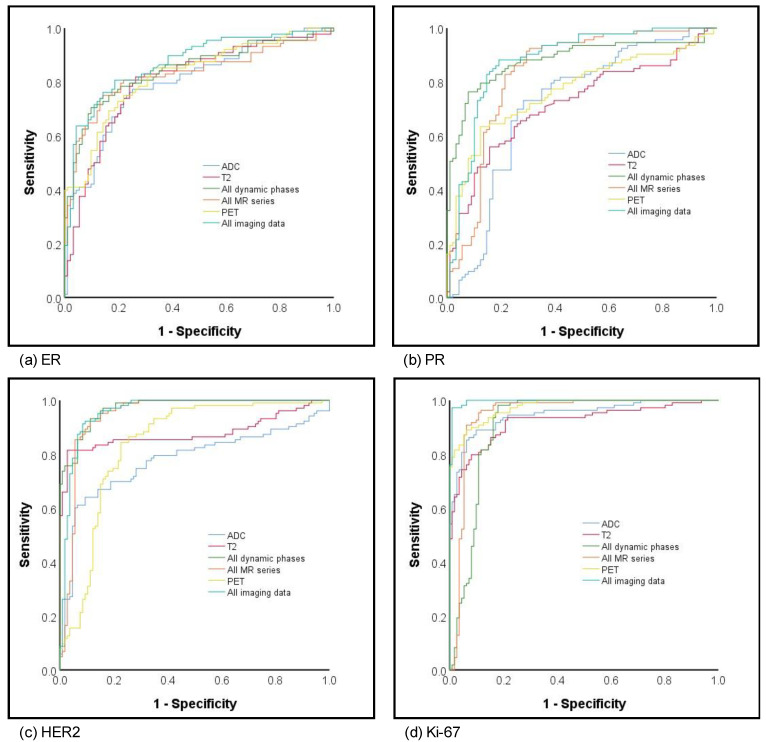
ROC curves for hormone (**a**) estrogene and (**b**) progesterone receptor status, (**c**) HER2 and (**d**) proliferation rate prediction.

The best results in terms of AUCs for prediction of ER status were achieved by a combination of all MR sequences and PET (0.87) followed by dynamic imaging only (all DCE) without PET, T2 or ADC, comprising an AUC of 0.84 and highest NPV of 88.1. Comparably, the highest AUC for prediction of PR status was also achieved based on all MR sequences combined with PET (AUC 0.88), followed by all dynamics (AUC 0.84). The highest AUCs > 0.90 and corresponding accuracies for prediction of HER2 status were achieved by all DCE (AUC 0.97; accuracy 89%) with comparable values achieved by all MR with (AUC 0.96; accuracy 89%) and without PET (AUC 0.95; accuracy 88.5%). Overall, the highest AUCs of all assessed parameters were achieved for prediction of Ki-67 with eight out of 10 imaging datasets yielding AUCs of ≥ 0.90 and the best results for all MR and PET with an AUC of 1.00 and 95.9% accuracy. Please see Figure 3 for corresponding AUC curves.

### 3.4. Radiomics Analysis to Predict Grading and Metastatic Disease

**Figure 4:**
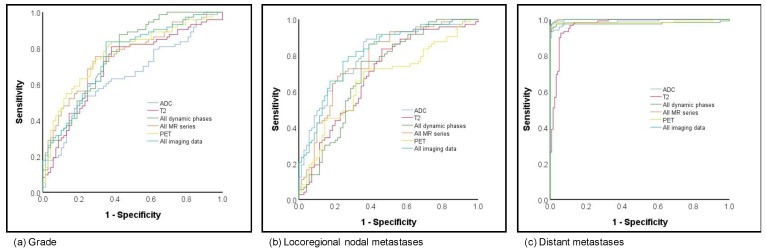
ROC curves for (**a**) grading and (**b**) local as well as (**c**) distant metastatic disease prediction.

Prediction of grading was analyzed as grade 1 versus grade 2 versus grade 3. When compared to prediction of subtype and hormonal status, the results for the prediction of grading were fair to moderate, with AUCs ranging from 0.65 to 0.78. The best and overall comparable results for prediction were achieved based on all dynamics, all MR and PET as well as all MR and PET (respective AUCs 0.76, 0.76, 0.77 and 0.75; Figure 4a).

Fair to moderate results were achieved for prediction of lymph node metastases, which was classified as negative lymph node versus positive. While the lowest AUCs were shown for the late phase dynamic phase (AUC 0.58), the highest AUCs, with 0.80, were based on the combined information of all MR and PET with comparable results for ADC imaging only (0.80; see Figure 4b). Considering an overall small number of patients with distant metastases (*n* = 7), all analyzed imaging sets with or without PET achieved excellent AUCs > 0.96 (see Figure 4c).

## 4. Discussion

In this study, we assessed the performance of a radiomics-based algorithmic analysis of simultaneous ^18^F-FDG PET/MRI datasets for non-invasive prediction of breast cancer phenotyping and tumor decoding. Our results underline the strength of multiparametric ^18^F-FDG PET/MRI as a valuable platform to determine an extensive set of imaging biomarkers of breast cancer. Furthermore, while our findings indicate that the combined analysis of multiparametric MRI and PET data provides the highest accuracy and predictive power for the most assessed imaging biomarkers, the results also underline the strength of multiparametric MRI only without PET to generate comparable accuracies.

Improved understanding of the association of tumor heterogeneity and personalized therapy of breast cancer has induced a transition from the previously mainly pathology-driven classification to a molecular level of phenotyping and tumor decoding. The importance of understanding tumor heterogeneity, the associated discrepancy of clinical subtype and molecular classification and the corresponding challenges of optimal treatment definition were underlined in recently published results of the Neoadjuvant Breast Registry Symphony Trial (NBRST). As tumors were classified by gene expression array with the molecular subtyping profile BluePrint as well as the MammaPrint prognostic profile, direct comparisons of treatment response according to conventional clinical versus molecular classification could be performed. With almost 20% of clinical “luminal“ patients being re-classified in a different subgroup, the results highlight the importance of MammaPrint/BluePrint for accurate identification of subtype biology and correct allocation of effective treatment to appropriate patients [31].

Over the past few years, a number of publications have investigated the potential of radiomics-based analyses for determination of different genomic and phenotypic characteristics of breast cancer as well as more fundamental features such as grading or metastatic spread [9,10,32]. Leithner et al. published a study focused exclusively on DCE-MRI for the differentiation of two important tumor characteristics, by means of molecular subtype and receptor status. According to their data, the best results for differentiation of luminal A versus luminal B amounted to an accuracy of 84.2%. Even better results were achieved in a more recent study by Leithner et al. where the radiomic analysis was exclusively based on diffusion weighted imaging or ADC data (apparent diffusion coefficient) [33]. Similarly, our results also showed an excellent accuracy of 92.3% based on all DCE-MRI sets, which could be further improved to an accuracy of 97.3%, when T2 weighted imaging and ADC were added into the analysis. Aside from the molecular subtype, the receptor status is another important factor for therapy management of breast cancer. While Leithner et al. chose a broader approach (hormone receptor positive versus negative) with rather limited success of determination (accuracy 68.1%), Li et al. recently published results which are comparable to ours. Our analysis yielded good differentiation of ER and PR positivity, reaching similar AUC values of 0.87 for ER (0.89, Li et al.) and 0.88 for PR [3]. Considering the different treatment options of breast cancer in accordance with their hormone receptor status and molecular subtype with varying associations with risk factors for incidence, response to treatment and risk of disease progression, our results demonstrate the potential for non-invasive whole-tumor tissue classification. Two further known prognostic factors associated with tumor aggressiveness, proliferative activity, disease spread rates and risk of recurrence are human epidermal growth factor receptor 2 (HER2) and Ki-67 overexpression [34,35]. Hence, pretherapeutic determination of HER2 and Ki-67 values is crucial for treatment planning, in particular regarding the administration of HER2 protein-targeting drugs. In our study, two datasets achieved similarly high results, (1) all DCE and (2) all MR and PET. Both datasets showed excellent AUCs of 0.97 (all DCE) and 0.96 (all MR and PET), with an equivalently high diagnostic accuracy of 89%. Bitencourt et al. recently published data on 311 patients, revealing even higher accuracy rates of 97.4% when combining MRI and clinical features for analysis [35]. This improvement in results after the inclusion of age, estrogen receptor status, lesion type and tumor size underlines the importance of clinical features. Comparable to a recent publication by Fan et al., our results for determination of Ki-67 overexpression underline the strength of utilizing multiparametric data [34]. Our results reveal a continuous increase in AUC values when adding T2 and ADC data (AUC 0.95) to exclusive DCE data (AUC 0.91), which is further improved when metabolic information based on PET is added (AUC 0.99). This indicates that different radiomic signatures are embedded in different morphologic, functional and metabolic datasets and a more comprehensive imaging platform may enable more precise analyses. Our results underline that non-invasive breast cancer phenotyping and tumor decoding prior to treatment are feasible. Whereas pre-treatment biopsy can only provide a snapshot of tumor biology and thus might not be representative of the molecular tumor heterogeneity, potentially causing therapy resistance and treatment failure, simultaneous ^18^F-FDG PET/MRI comprehensive radiomics analysis can overcome this limitation through non-invasive whole-tumor assessment. More accurate tumor phenotyping is particularly relevant in the setting of the increased use of neo-adjuvant cytotoxic, endocrine and targeted therapies, as tumor biology might be subject to change over time and with treatment, and monitoring under therapy can provide indicators for efficient treatment adaptation when needed [36].

Apart from more recently introduced genomic and phenotypic characteristics, three features, grading, lymphonodular status and distant metastatic spread, are well-established biomarkers for prognosis. Comparable to previous publications, our prediction results for grading and lymph node metastases fell short in their diagnostic accuracy [8,34]. Similar to previously published results by Demircioglu et al. from an MRI-based study, our results for grading amounted to an AUC of 0.78 (0.74, respectively) and 0.80 for lymph node metastases (0.71) [8]. Only the determination of distant metastases achieved excellent AUC values of 0.96. While these results may in part result from the lack of radiomic signatures in the assessed imaging data for these particular characteristics, they may also be biased due to a heterogeneous and uneven patient cohort, reflected in the small number of patients with grade 1 tumors as well as distant metastases.

Whilst radiomics-based analyses of breast cancer have become a well-investigated research focus over the past few years, the majority of studies were based either on mammographic or MR-based imaging [3,8,34,35,37]. Only a few studies included PET-based data in their analysis, with Krajnc et al. and Huang et al. demonstrating promising results in recent publications [13,38]. Krajnc et al. used ^18^F-FDG PET/CT imaging combined with data preprocessing and radiomics analysis to characterize breast tumors. Notably, their predictive models achieved good results in breast cancer detection (AUC 0.82) and identification of triple-negative tumors (AUC 0.82), yet determination of luminal A/B subtype and the individual receptor status yielded low performance, with AUCs ranging from 0.46–0.68 [13]. Their results underline the potential of PET-based metabolic data for radiomic signature derivation and indicate that CT-based data may not provide a sufficiently comprehensive platform for breast cancer assessment. This potential shortage was addressed in a study by Huang et al. who used retrospectively fused PET- and MRI-derived features to decode breast cancer phenotypes and prognosis [14]. Unsupervised clustering based on PET and MRI radiomic features created three subgroups which showed significant associations with tumor grade, overall stage, subtypes and disease recurrence [14]. While their analyses revealed promising and partially comparable results for tumor grading and subtype differentiation to our study, two important differences lie in the retrospective fusion of PET and MRI data (versus simultaneous acquisition) and the exclusive focus on DCE-MRI data (versus additional T2 or diffusion weighted imaging as presented in our study).

While our study reveals promising results regarding the potential of ^18^F-FDG PET/MRI as a platform for radiomics-based analyses of breast cancer, limitations of the current study should be addressed in future trials. Small numbers in the minority class for some outcomes, including luminal A cases, suggest that these results need to be regarded as preliminary. Radiomics studies are known to benefit from large patient cohorts as well as multicenter analyses, hence these two important aspects should be approached in prospective study set-ups.

## 5. Conclusions

To the best of our knowledge, our study is the first to demonstrate that simultaneous ^18^F-FDG PET/MRI facilitates a comprehensive platform for highly accurate, non-invasive tumor phenotyping and decoding. Although radiogenomics-based tissue analysis is unlikely to replace invasive tissue sampling in the foreseeable future, it bears the potential to provide imaging biomarkers as auxiliary parameters for patient stratification. Radiogenomic characteristics derived from multiparametric PET/MRI studies may promote understanding and therapy monitoring of tumor biology of the whole tumor instead of focal invasive tissue sampling which is known to provide erroneous and inaccurate assessment. With increased understanding of the importance of correct subtyping of breast cancer in regard to chemosensitivity [39], it will be interesting to see whether imaging-based analysis will be able to measure up to multigene classifier testing and potentially even add value due to whole-tumor analysis in the future.

Overall, when putting our results into perspective with previous publications on MRI-, PET/CT- or retrospectively fused PET/MRI-based studies, it becomes apparent that some assessed tumor characteristics seem to benefit from the added information of simultaneously obtained multiparametric MRI and PET data. Nevertheless, our results also underline the potential and strength of multiparametric MRI data only for high-quality radiomics analysis of breast cancer.

## Figures and Tables

**Table 1:** Patients characteristics.

Total Patients		124 (Mean Age 54 y; Range 31–86 y)
Menopause Status		
	Pre	55 (44%)
	Peri	12 (10%)
	Post	57 (46%)
Tumor Volume (cm^3^)—Median (IQR)		7.27 (3.29–13.74)
Histologic Subtype		
	NST	109 (88%)
	Lobular invasive	7 (6%)
	other	8 (6%)
Molecular Subtype		
	Luminal A	17 (14%)
	Luminal B	82 (66%)
	HER2-enriched	5 (4%)
	Triple negative	19 (16%)
Ki-67		Mean: 40, range 3–97%
	Negative (<15%)	13 (10%)
	Positive (>15%)	111 (90%)
Tumor Grade		
	G1	5 (4%)
	G2	67 (54%)
	G3	52 (42%)
N-status		
	Positive	49 (40%)
	Negative	75 (60%)
M-status		
	Positive	7 (6%)
	Negative	117 (94%)

**Table 2:** Selection of best mean classification accuracies achieved for prediction of each assessed imaging biomarker.

Radiomics Analysis to Predict	Best Results by	AUC	Sensitivity	Specificity	PPV	NPV	Accuracy
Subtype (luminal A versus luminal B)	All MR	0.978 (0.950–1.000)	94.6 (87.9–98.2)	100.0 (96.0–100.0)	100.0 (95.9–100.0)	94.7 (88.1–98.3)	97.3 (93.7–99.1)
Subtype (luminals vs. others)	PET	0.950 (0.922–0.979)	83.5 (74.6–90.3)	93.2 (86.5–97.2)	92.0 (84.3–96.7)	85.7 (77.8–91.6)	88.5 (83.2–92.6)
ER Status (negative vs. positive)	All MR and PET	0.870 (0.818–0.923)	90.1 (82.1–95.4)	65.9 (55.0–75.7)	73.2 (64.0 -81.1)	86.6 (76.0–93.7)	78.2 (71.4–84.0)
PR Status (negative vs. positive)	All MR and PET	0.879 (0.826–0.932)	84.1 (74.8–91.0)	83.9 (74.8–90.7)	83.1 (73.7–90.2)	84.8 (75.8–91.4)	84.0 (77.8–89.0)
HER2 (negative vs. positive)	All DCE	0.972 (0.955–0.989)	84.9 (76.6–91.1)	93.2 (86.5–97.2)	92.8 (85.7–97.0)	85.7 (77.8–91.6)	89.0 (83.9–92.9)
Proliferation (high vs. low)	All MR and PET	0.997 (0.992–1.000)	99.1 (95.1–100.0)	92.7 (86.0–96.8)	93.2 (87.1–97.0)	99.0 (94.7–100.0)	95.9 (92.4–98.1)
Grade (grade 1 vs. grade 2 vs. grade 3)	PET	0.771 (0.693–0.849)	66.2 (53.7–77.2)	78.1 (66.9–86.9)	73.8 (60.9–84.2)	71.3 (60.0–80.8)	72.3 (64.2–79.5)
Nodal Status (0 vs. 1, 2, 3)	All MR and PET	0.810 (0.740–0.881)	63.8 (51.3–75.0)	82.2 (71.5–90.2)	77.2 (64.2–87.3)	70.6 (59.7–80.0)	73.2 (65.2–80.3)
Distant Metastases (0 vs. 1)	All MR and PET	0.999 0.997–1.000)	98.3 (94.0–99.8)	98.3 (94.0–99.8)	98.3 (94.0–99.8)	98.3 (94.0–99.8)	98.3 (95.7–99.5)

## Data Availability

The data presented in this study are available on request from the corresponding author. The data are not publicly available due to privacy restrictions.

## Supplementary Materials

The following are available online at https://www.mdpi.com/article/10.3390/cancers13122928/s1, Table S1: Classification accuracies achieved for prediction of each assessed imaging biomarker for Luminal A verus Luminal B; Table S2: Classification accuracies achieved for prediction of each assessed imaging biomarker for Luminals vs Others, Table S3: Classification accuracies achieved for prediction of each assessed imaging biomarker for ER Status, Table S4: Classification accuracies achieved for prediction of each assessed imaging biomarker for PR status, Table S5: Classification accuracies achieved for prediction of each assessed imaging biomarker for HER2 status, Table S6: Classification accuracies achieved for prediction of each assessed imaging biomarker for Ki-67, Table S7: Classification accuracies achieved for prediction of each assessed imaging biomarker for Grading, Table S8: Classification accuracies achieved for prediction of each assessed imaging biomarker for Nodal Status, Table S9: Classification accuracies achieved for prediction of each assessed imaging biomarker for distant metastases, Table S10: Subtype (Luminal A vs. Luminal B), Table S11: Subtype (luminals vs others), Table S12: ER Status (negative vs. positive), Table S13: PR status (negative vs. positive), Table S14: HER2 status (negative vs. positive), Table S15: Proliferation (high vs. low), Table S16: Grade (grade 1 and 2 vs. Grade 3), Table S17: Nodal Status (0 vs. 1,2,3), Table S18: Metastases (0 vs. 1).
