# Supplementary material for: Multiparametric Integrated 18F-FDG PET/MRI-Based Radiomics for Breast Cancer Phenotyping and Tumor Decoding
Source: Cancers (Basel). 2021 Jun 11;13(12):2928. doi: 10.3390/cancers13122928 (PMC8230865; doi:10.3390/cancers13122928)
Supplement: Supplementary file 1 [file cancers-13-02928-s001.zip › cancers-1228752-SI.pdf]

Supplementary Materials: Multiparametric Integrated ^{18}F-FDG PET/MRI-based Radiomics for Breast Cancer Phenotyping and Tumor Decoding

Lale Umutlu, Julian Kirchner, Nils Martin Bruckmannn, Janna Morawitz, Gerald Antoch, Marc Ingenwerth, Ann-Kathrin Bittner, Oliver Hoffmann, Johannes Haubold, Johannes Grueneisen, Harald H. Quick, Christoph Rischpler, Ken Herrmann, Peter Gibbs and Katja Pinker-Domenig

Table S1. Classification accuracies achieved for prediction of each assessed imaging biomarker for Luminal A verus Luminal B.

Images	AUC	Sensitivity	Specificity	PPV	NPV	Accuracy
ADC	0.976<br>(0.952–1.000)	96.8<br>(90.9–99.3)	90.0<br>(81.9–95.3)	90.9<br>(83.4–95.8)	96.4<br>(89.9–99.3)	93.4<br>(88.8–96.6)
T2	0.821<br>(0.755–0.887)	78.5<br>(68.8–86.3)	77.8<br>(67.8–85.9)	78.5<br>(68.8–86.3)	77.8<br>(67.8–85.9)	78.1<br>(71.4–83.9)
Dynamic 1	0.921<br>(0.876–0.966)	89.2<br>(81.1–94.7)	86.7<br>(77.9–92.9)	87.4<br>(79.0–93.3)	88.6<br>(80.1–94.4)	88.0<br>(82.4–92.3)
Dynamic 2	0.951<br>(0.920–0.983)	88.2<br>(79.8–93.9)	91.1<br>(83.2–96.1)	91.1<br>(83.2–96.1)	88.2<br>(79.8–93.9)	89.6<br>(84.3–93.6)
Dynamic 3	0.924<br>(0.885–0.963)	83.9<br>(74.8–90.7)	86.7<br>(77.9–92.9)	86.7<br>(77.9–92.9)	83.9<br>(74.8–90.7)	85.2<br>(79.3–90.0)
Dynamic 4	0.934<br>(0.896–0.971)	80.6<br>(71.1–88.1)	91.1<br>(83.2–96.1)	90.4<br>(81.9–95.7)	82.0<br>(73.1–89.0)	85.8<br>(79.9–90.5)
Dynamic 5	0.967<br>(0.936–0.997)	92.5<br>(85.1–96.9)	94.4<br>(87.5–98.2)	94.5<br>(87.6–98.2)	92.4<br>(84.9–96.9)	93.4<br>(88.8–96.6)
All Dynamics	0.983<br>(0.968–0.999)	97.8<br>(92.4–99.7)	86.7<br>(77.9–92.9)	88.3<br>(80.5–93.8)	97.5<br>(91.3–99.7)	92.3<br>(87.5–95.8)
All MR	0.978<br>(0.950–1.000)	94.6<br>(87.9–98.2)	100.0<br>(96.0–100.0)	100.0<br>(95.9–100.0)	94.7<br>(88.1–98.3)	97.3<br>(93.7–99.1)
PET	0.944<br>(0.909–0.979)	89.2<br>(81.1–94.7)	88.9<br>(80.5–94.5)	89.2<br>(81.1- 94.7)	88.9<br>(80.5–94.5)	89.1<br>(83.6–93.2)
All MR and PET	0.980<br>(0.953–1.000)	92.5<br>(85.1–96.9)	100.0<br>(96.0–100.0)	100.0<br>(95.8–100.0)	92.8<br>(85.7–97.0)	96.2<br>(92.3–98.4)

Table S2. Classification accuracies achieved for prediction of each assessed imaging biomarker for Luminals vs. Others.

Images	AUC	Sensitivity	Specificity	PPV	NPV	Accuracy
ADC	0.846<br>(0.791–0.901)	81.4<br>(72.3–88.6)	74.8<br>(65.2–82.8)	75.2<br>(65.9–83.1)	81.1<br>(71.7–88.4)	78.0<br>(71.6–83.5)
T2	0.872<br>(0.821–0.922)	70.1<br>(60.0–79.0)	85.4<br>(77.1–91.6)	81.9<br>(72.0–89.5)	75.2<br>(66.4–82.7)	78.0<br>(71.6–83.5)
Dynamic 1	0.809<br>(0.747–0.871)	68.0<br>(57.8–77.1)	80.6<br>(71.6–87.7)	76.7<br>(66.4–85.2)	72.8<br>(63.7–80.7)	74.5<br>(67.9–80.4)
Dynamic 2	0.853<br>(0.797–0.909)	78.4<br>(68.8–86.1)	80.6<br>(71.6–87.7)	79.2<br>(69.7–86.8)	79.8<br>(70.8–87.0)	79.5<br>(73.2–84.9)
Dynamic 3	0.862<br>(0.807–0.918)	87.6<br>(79.4–93.4)	75.7<br>(66.3–83.6)	77.3<br>(68.3–84.7)	86.7<br>(77.9–92.9)	81.5<br>(75.4–86.6)
Dynamic 4	0.878<br>(0.825–0.930)	77.3<br>(67.7–85.2)	90.3<br>(82.9–95.2)	88.2<br>(79.4–94.2)	80.9<br>(72.5–87.6)	84.0<br>(78.2–88.8)
Dynamic 5	0.915<br>(0.873–0.957)	79.4<br>(70.0–86.9)	89.3<br>(81.7–94.5)	87.5<br>(78.7–93.6)	82.1<br>(73.8–88.7)	84.5<br>(78.7–89.2)
All Dynamics	0.919<br>(0.876–0.961)	81.4<br>(72.3–88.6)	92.2<br>(85.3–96.6)	90.8<br>(82.7–95.9)	84.1<br>(76.0–90.3)	87.0<br>(81.5–91.3)
All MR	0.917	80.4	89.3	87.6	82.9	85.0

	(0.876–0.959)	(71.1–87.8)	(81.7–94.5)	(79.0–93.7)	(74.6–89.4)	(79.3–89.6)
PET	0.950 (0.922–0.979)	83.5 (74.6–90.3)	93.2 (86.5–97.2)	92.0 (84.3–96.7)	85.7 (77.8–91.6)	88.5 (83.2–92.6)
All MR and PET	0.930 (0.893–0.966)	92.8 (85.7–97.0)	83.5 (74.9–90.1)	84.1 (75.8–90.5)	92.5 (85.1–96.9)	88.0 (82.7–92.2)

Table S3. Classification accuracies achieved for prediction of each assessed imaging biomarker for ER Status.

Images	AUC	Sensitivity	Specificity	PPV	NPV	Accuracy
ADC	0.799 (0.734–0.864)	82.4 (73.0–89.6)	63.6 (52.7–73.6)	70.1 (60.5–78.6)	77.8 (66.4–86.7)	73.2 (66.1–79.5)
T2	0.805 (0.740–0.871)	78.0 (68.1–86.0)	73.9 (63.4–82.7)	75.5 (65.6–83.8)	76.5 (66.0–85.0)	76.0 (69.0–82.0)
Dynamic 1	0.808 (0.744–0.871)	76.9 (66.9–85.1)	70.5 (59.8–79.7)	72.9 (62.9–81.5)	74.7 (64.0–83.6)	73.7 (66.7–80.0)
Dynamic 2	0.825 (0.766–0.884)	68.1 (57.5–77.5)	76.1 (65.9–84.6)	74.7 (64.0–83.6)	69.8 (59.6–78.7)	72.1 (64.9–78.5)
Dynamic 3	0.733 (0.659–0.808)	63.7 (53.0–73.6)	78.4 (68.4–86.5)	75.3 (64.2–84.4)	67.6 (57.7–76.6)	70.9 (63.7–77.5)
Dynamic 4	0.786 (0.720–0.852)	71.4 (61.0–80.4)	73.9 (63.4–82.7)	73.9 (63.4–82.7)	71.4 (61.0–80.4)	72.6 (65.5–79.0)
Dynamic 5	0.635 (0.552–0.717)	62.6 (51.9–72.6)	65.9 (55.0–75.7)	65.5 (54.6–75.4)	63.0 (52.3–72.9)	64.2 (56.8–71.3)
All Dynamics	0.844 (0.785–0.904)	91.2 (83.4–96.1)	67.0 (56.2–76.7)	74.1 (65.0–81.9)	88.1 (77.8–94.7)	79.3 (72.7–85.0)
All MR	0.829 (0.765–0.892)	86.8 (78.1–93.0)	70.5 (59.8–79.7)	75.2 (65.9–83.1)	83.8 (73.4–91.3)	78.8 (72.0–84.5)
PET	0.826 0.766–0.886)	80.2 (70.6–87.8)	71.6 (61.0–80.7)	74.5 (64.7–82.8)	77.8 (67.2–86.3)	76.0 (69.0–82.0)
All MR and PET	0.870 (0.818–0.923)	90.1 (82.1–95.4)	65.9 (55.0–75.7)	73.2 (64.0 -81.1)	86.6 (76.0–93.7)	78.2 (71.4–84.0)

Table S4. Classification accuracies achieved for prediction of each assessed imaging biomarker for PR status.

Images	AUC	Sensitivity	Specificity	PPV	NPV	Accuracy
ADC	0.715 (0.637–0.793)	62.5 (51.5–72.6)	77.4 (67.6–85.4)	72.4 (60.9–82.0)	68.6 (58.8–77.3)	70.2 (62.9–76.7)
T2	0.718 (0.643–0.794)	71.6 (61.0–80.7)	65.6 (55.0–75.1)	66.3 (55.9–75.7)	70.9 (60.1–80.2)	68.5 (61.2–75.2)
Dynamic 1	0.797 (0.731–0.864)	79.5 (69.6–87.4)	71.0 (60.6–79.9)	72.2 (62.1–80.8)	78.6 (68.3–86.8)	75.1 (68.2–81.3)
Dynamic 2	0.607 (0.525–0.689)	54.5 (43.6–65.2)	62.4 (51.7–72.2)	57.8 (46.5–68.6)	59.2 (48.8–69.0)	58.6 (51.0–65.8)
Dynamic 3	0.739 (0.665–0.813)	64.8 (53.9–74.7)	77.4 (67.6–85.4)	73.1 (61.8–82.5)	69.9 (60.1–78.5)	71.3 (64.1–77.7)
Dynamic 4	0.788 (0.722–0.854)	77.3 (67.1–85.5)	67.7 (57.3–77.1)	69.4 (59.3–78.3)	75.9 (65.3–84.6)	72.4 (65.3–78.7)
Dynamic 5	0.790 (0.724–0.856)	73.9 (63.4–82.7)	76.3 (66.4–84.5)	74.7 (64.3–83.4)	75.5 (65.6–83.8)	75.1 (68.2–81.3)
All Dynamics	0.881 (0.828–0.934)	87.5 (78.7–93.6)	78.5 (68.8–86.3)	79.4 (70.0–86.9)	86.9 (77.8–93.3)	82.9 (76.6–88.1)
All MR	0.831 (0.767–0.895)	75.0 (64.6–83.6)	86.0 (77.3–92.3)	83.5 (73.5–90.9)	78.4 (69.2–86.0)	80.7 (74.1–86.1)
PET	0.767 (0.697–0.838)	80.7 (70.9–88.3)	64.5 (53.9–74.2)	68.3 (58.4–77.1)	77.9 (67.0–86.6)	72.4 (65.3–78.7)
All MR and PET	0.879 (0.826–0.932)	84.1 (74.8–91.0)	83.9 (74.8–90.7)	83.1 (73.7–90.2)	84.8 (75.8–91.4)	84.0 (77.8–89.0)

Table S5. Classification accuracies achieved for prediction of each assessed imaging biomarker for HER2 status.

Images	AUC	Sensitivity	Specificity	PPV	NPV	Accuracy
ADC	0.777<br>(0.710–0.845)	86.8<br>(78.8–92.6)	64.1<br>(54.0–73.3)	71.3<br>(62.7–78.9)	82.5<br>(72.4–90.1)	75.6<br>(69.2–81.3)
T2	0.878<br>(0.825–0.932)	94.3<br>(88.1–97.9)	81.6<br>(72.7–88.5)	84.0<br>(76.2–90.1)	93.3<br>(86.1–97.5)	88.0<br>(82.9–92.1)
Dynamic 1	0.871<br>(0.823–0.919)	74.5<br>(65.1–82.5)	87.4<br>(79.4–93.1)	85.9<br>(77.0–92.3)	76.9<br>(68.2–84.5)	80.9<br>(74.9–86.0)
Dynamic 2	0.795<br>(0.733–0.857)	67.9<br>(58.2–76.7)	84.5<br>(76.0–90.9)	81.8<br>(72.2–89.2)	71.9<br>(63.0–79.7)	76.1<br>(69.7–81.7)
Dynamic 3	0.853<br>(0.800–0.905)	77.4<br>(68.2–84.9)	81.6<br>(72.7–88.5)	81.2<br>(72.2–88.3)	77.8<br>(68.8–85.2)	79.4<br>(73.3–84.7)
Dynamic 4	0.656<br>(0.582–0.730)	60.4<br>(50.4–69.7)	64.1<br>(54.0–73.3)	63.4<br>(53.2–72.7)	61.1<br>(51.3–70.3)	62.2<br>(55.3–68.8)
Dynamic 5	0.876<br>(0.826–0.925)	81.1<br>(72.4–88.1)	85.4<br>(77.1–91.6)	85.1<br>(76.7–91.4)	81.5<br>(72.9–88.3)	83.3<br>(77.5–88.0)
All Dynamics	0.972<br>(0.955–0.989)	84.9<br>(76.6–91.1)	93.2<br>(86.5–97.2)	92.8<br>(85.7–97.0)	85.7<br>(77.8–91.6)	89.0<br>(83.9–92.9)
All MR	0.943<br>(0.907–0.979)	81.1<br>(72.4–88.1)	96.1<br>(90.4–98.9)	95.6<br>(89.0–98.8)	83.2<br>(75.2–89.4)	88.5<br>(83.4–92.5)
PET	0.839<br>(0.782–0.896)	77.4<br>(68.2–84.9)	84.5<br>(76.0–90.9)	83.7<br>(74.8–90.4)	78.4<br>(69.6–85.6)	80.9<br>(74.9–86.0)
All MR and PET	0.959<br>(0.931–0.986)	81.1<br>(72.4–88.1)	97.1<br>(91.7–99.4)	96.6<br>(90.5–99.3)	83.3<br>(75.4–89.5)	89.0<br>(83.9–92.9)

Table S6. Classification accuracies achieved for prediction of each assessed imaging biomarker for Ki-67.

Images	AUC	Sensitivity	Specificity	PPV	NPV	Accuracy
ADC	0.947<br>(0.919–0.976)	91.0<br>(84.1–95.6)	87.2<br>(79.4–92.8)	87.8<br>(80.4 -93.2)	90.5<br>(83.2–95.3)	89.1<br>(84.2–92.9_
T2	0.922<br>(0.884–0.959)	90.1<br>(83.0–94.9)	79.8<br>(71.1–86.9)	82.0<br>(74.0–88.3)	88.8<br>(80.8–94.3)	85.0<br>(79.6–89.4)
Dynamic 1	0.878<br>(0.830–0.926)	73.0<br>(63.7–81.0)	93.6<br>(87.2–97.4)	92.0<br>(84.3–96.7)	77.3<br>(69.2–84.1)	83.2<br>(77.6–87.9)
Dynamic 2	0.850<br>(0.795–0.905)	70.3<br>(60.9–78.6)	93.6<br>(87.2–97.4)	91.8<br>(83.8–96.6)	75.6<br>(67.4–82.5)	81.8<br>(76.1–86.7)
Dynamic 3	0.894<br>(0.844–0.943)	78.4<br>(69.6–85.6)	96.3<br>(90.9–99.0)	95.6<br>(89.1–98.8)	81.4<br>(73.6–87.7)	87.3<br>(82.1–91.4)
Dynamic 4	0.907<br>(0.864–0.949)	84.7<br>(76.6–90.8)	79.8<br>(71.1–86.9)	81.0<br>(72.7–87.7)	83.7<br>(75.1–90.2)	82.3<br>(76.6–87.1)
Dynamic 5	0.946<br>(0.919–0.973)	89.2<br>(81.9–94.3)	82.6<br>(74.1–89.2)	83.9<br>(76.0–90.0)	88.2<br>(80.4–93.8)	85.9<br>(80.6–90.2)
All Dynamics	0.911<br>(0.867–0.955)	81.1<br>(72.5–87.9)	98.2<br>(93.5–99.8)	97.8<br>(92.4–99.7)	83.6<br>(76.0–89.5)	89.5<br>(84.7–93.3)
All MR	0.947<br>(0.911–0.983)	83.8<br>(75.6–90.1)	97.2<br>(92.2–99.4)	96.9<br>(91.1–99.4)	85.5<br>(78.0–91.2)	90.5<br>(85.8–94.0)
PET	0.975<br>(0.960–0.990)	86.5<br>(78.7–92.2)	91.7<br>(84.9–96.2)	91.4<br>(84.4–96.0)	87.0<br>(79.4–92.5)	89.1<br>(84.2–92.9)
All MR and PET	0.997<br>(0.992–1.000)	99.1<br>(95.1–100.0)	92.7<br>(86.0–96.8)	93.2<br>(87.1–97.0)	99.0<br>(94.7–100.0)	95.9<br>(92.4–98.1)

Table S7. Classification accuracies achieved for prediction of each assessed imaging biomarker for Grading.

Images	AUC	Sensitivity	Specificity	PPV	NPV	Accuracy
ADC	0.648<br>(0.558–0.739)	60.3<br>(47.7–72.0)	63.0<br>(50.9–74.0)	60.3<br>(47.7–72.0)	63.0<br>(50.9–74.0)	61.7<br>(53.1–69.8)

T2	0.705 (0.618–0.792)	63.2 (50.7–74.6)	75.3 (63.9–84.7)	70.5 (57.4–81.5)	68.8 (57.4–78.7)	69.5 (61.2–77.0)
Dynamic 1	0.567 (0.471–0.663)	38.2 (26.7–50.8)	79.5 (68.4–88.0)	63.4 (46.9–77.9)	58.0 (47.7–67.8)	59.6 (51.0–67.7)
Dynamic 2	0.648 (0.556–0.739)	48.5 (36.2–61.0)	78.1 (66.9–86.9)	67.3 (52.5–80.1)	62.0 (51.2–71.9)	63.8 (55.3–71.7)
Dynamic 3	0.704 (0.620–0.789)	48.5 (36.2–61.0)	84.9 (74.6–92.2)	75.0 (59.7–86.8)	63.9 (53.5–73.4)	67.4 (59.0–75.0)
Dynamic 4	0.648 (0.555–0.741)	48.5 (36.2–61.0)	76.7 (65.4–85.8)	66.0 (51.2–78.8)	61.5 (50.8–71.6)	63.1 (54.6–71.1)
Dynamic 5	0.654 (0.562–0.745)	52.9 (40.4–65.2)	78.1 (66.9–86.9)	69.2 (54.9–81.3)	64.0 (53.2–73.9)	66.0 (57.5–73.7)
All Dynamics	0.762 (0.684–0.840)	55.9 (43.3–67.9)	87.7 (77.9–94.2)	80.9 (66.7–90.9)	68.1 (57.7–77.3)	72.3 (64.2–79.5)
All MR	0.761 (0.683–0.839)	70.6 (58.3–81.0)	75.3 (63.9–84.7)	72.7 (60.4–83.0)	73.3 (61.9–82.9)	73.0 (64.9–80.2)
PET	0.771 (0.693–0.849)	66.2 (53.7–77.2)	78.1 (66.9–86.9)	73.8 (60.9–84.2)	71.3 (60.0–80.8)	72.3 (64.2–79.5)
All MR and PET	0.750 (0.670–0.830)	63.2 (50.7–74.6)	83.6 (73.0–91.2)	78.2 (65.0–88.2)	70.9 (60.1–80.2)	73.8 (65.7–80.8)

Table S8. Classification accuracies achieved for prediction of each assessed imaging biomarker for Nodal Status.

Images	AUC	Sensitivity	Specificity	PPV	NPV	Accuracy
ADC	0.805 (0.733–0.877)	68.1 (55.8–78.8)	75.3 (63.9–84.7)	72.3 (59.8–82.7)	71.4 (60.0–81.2)	71.8 (63.7–79.1)
T2	0.694 (0.606–0.782)	53.6 (41.2–65.7)	82.2 (71.5–90.2)	74.0 (59.7–85.4)	65.2 (54.6–74.9)	68.3 (60.0–75.9)
Dynamic 1	0.638 (0.547–0.730)	66.7 (54.3–77.6)	57.5 (45.4–69.0)	59.7 (47.9–70.8)	64.6 (51.8–76.1)	62.0 (53.5–70.0)
Dynamic 2	0.712 (0.628–0.795)	62.3 (49.8–73.7)	65.8 (53.7–76.5)	63.2 (50.7–74.6)	64.9 (52.9–75.6)	64.1 (55.6–72.0)
Dynamic 3	0.717 (0.634–0.801)	68.1 (55.8–78.8)	65.8 (53.7–76.5)	65.3 (53.1–76.1)	68.6 (56.4–79.1)	66.9 (58.5–74.6)
Dynamic 4	0.648 (0.558–0.737)	68.1 (55.8–78.8)	54.8 (42.7–66.5)	58.8 (47.2–69.6)	64.5 (51.3–76.3)	61.3 (52.7–69.3)
Dynamic 5	0.578 (0.481–0.675)	47.8 (35.6–60.2)	74.0 (62.4–83.5)	63.5 (49.0–76.4)	60.0 (49.1–70.2)	61.3 (52.7–69.3)
All Dynamics	0.706 (0.619–0.793)	52.2 (39.8–64.4)	80.8 (69.9–89.1)	72.0 (57.5–83.8)	64.1 (53.5–73.9)	66.9 (58.5–74.6)
All MR	0.777 (0.700–0.855)	60.9 (48.4–72.4)	86.3 (76.2–93.2)	80.8 (67.5–90.4)	70.0 (59.4–79.2)	73.9 (65.9–80.9)
PET	0.663 (0.573–0.753)	63.8 (51.3–75.0)	67.1 (55.1–77.7)	64.7 (52.2–75.9)	66.2 (54.3–76.8)	65.5 (57.1–73.3)
All MR and PET	0.810 (0.740–0.881)	63.8 (51.3–75.0)	82.2 (71.5–90.2)	77.2 (64.2–87.3)	70.6 (59.7–80.0)	73.2 (65.2–80.3)

Table S9. Classification accuracies achieved for prediction of each assessed imaging biomarker for distant metastases.

Images	AUC	Sensitivity	Specificity	PPV	NPV	Accuracy
ADC	0.988<br>(0.976–1.000)	97.4<br>(92.7–99.5)	94.1<br>(88.2–97.6)	94.2<br>(88.4–97.6)	97.4<br>(92.5–99.5)	95.7<br>(92.3–97.9)
T2	0.968<br>(0.946–0.990)	88.9<br>(81.7–93.9)	95.8<br>(90.4–98.6)	95.4<br>(89.6–98.5)	89.7<br>(83.0–94.4)	92.3<br>(88.2–95.4)
Dynamic 1	0.997<br>(0.993–1.000)	100.0<br>(96.9–100.0)	93.2<br>(87.1–97.0)	93.6<br>(87.8–97.2)	100.0<br>(96.7–100.0)	96.6<br>(93.4–98.5)
Dynamic 2	0.992<br>(0.986–0.999)	93.2<br>(87.0–97.0)	98.3<br>(94.0–99.8)	98.2<br>(93.6–99.8)	93.5<br>(87.2–97.2)	95.7<br>(92.3–97.9)
Dynamic 3	0.965<br>(0.940–0.991)	99.1<br>(95.3–100.0)	87.3<br>(79.9–92.7)	88.5<br>(81.8–93.4)	99.0<br>(94.8–100.0)	93.2<br>(89.2–96.1)
Dynamic 4	0.964<br>(0.934–0.994)	99.1<br>(95.3–100.0)	89.0<br>(81.9–94.0)	89.9<br>(83.4–94.5)	99.1<br>(94.9–100.0)	94.0<br>(90.2–96.7)
Dynamic 5	0.989<br>(0.974–1.000)	99.1<br>(95.3 -100.0)	90.7<br>(83.9–95.3)	91.3<br>(85.0–95.6)	99.1<br>(94.9–100.0)	94.9<br>(91.3–97.3)
All Dynamics	0.977<br>(0.952–1.000)	99.1<br>(95.3–100.0)	94.1<br>(88.2–97.6)	94.3<br>(88.6–97.7)	99.1<br>(94.9–100.0)	96.6<br>(93.4–98.5)
All MR	0.999<br>(0.997–1.000)	99.1<br>(95.3–100.0)	97.5<br>(92.7–99.5)	97.5<br>(92.8–99.5)	99.1<br>(94.9–100.0)	98.3<br>(95.7–99.5)
PET	0.986<br>(0.967–1.000)	100.0<br>(96.9–100.0)	93.2<br>(87.1–97.0)	93.6<br>(87.8–97.2)	100.0<br>(96.7–100.0)	96.6<br>(93.4–98.5)
All MR and PET	0.999<br>0.997–1.000)	98.3<br>(94.0–99.8)	98.3<br>(94.0–99.8)	98.3<br>(94.0–99.8)	98.3<br>(94.0–99.8)	98.3<br>(95.7–99.5)

Table S10. Subtype (Luminal A vs. Luminal B).

Images	1st	2nd	3rd	4th	5th	6th
ADC	cluster prominence (GLCM)	sum variance (GLCM)	kurtosis (FO)	glnNorm (SZM)	joint variance (GLCM)	maximum (FO)
T2	texture strength (NGTDM)	coarseness (NGTDM)	contrast (NGTDM)	total energy (FO)	joint maximum (GLCM)	complexity (NGTDM)
DCE 1	complexity (NGTDM)	srlgle (RLM)	glnNorm (SZM)	lzhgle (SZM)	joint maximum (GLCM)	lglze (SZM)
DCE 2	correlation (GLCM)	coeffVar (FO)	hdhge (NGLDM)	median (FO)	coeffDisp (FO)	joint maximum (GLCM)
DCE 3	coeffVar (FO)	hdhge (NGLDM)	gray level variance (NGLDM)	hglze (SZM)	dcv (NGLDM)	ldlge (NGLDM)
DCE 4	run emphasis (RLM)	gray level variance (NGLDM)	gray level variance (SZM)	interQuartRange (FO)	hdhge (NGLDM)	texture strength (NGTDM)
DCE 5	glnNorm (SZM)	kurtosis (FO)	auto correlation (GLCM)	lglze (SZM)	texture strength (NGTDM)	coarseness (NGTDM)
All DCE	szlgle (SZM P3)	glnNorm (SZM P4)	inverse variance (GLCM P4)	complexity (NGTDM P1)	hdhge (NGLDM P5)	entropy (FO P1)
All MR	complexity (NGTDM P1)	inverse variance (GLCM P4)	difference variance (GLCM P4)	minimum (FO T2)	joint maximum (GLCM T2)	secondInfCorr (GLCM ADC)
PET	interQuartRange (FO)	sum variance (GLCM)	range (FO)	cluster shade (GLCM)	entropy (FO)	lzlgle (SZM)
All MR and PET	inverse variance (GLCM P4)	complexity (NGTDM P1)	minimum (FO T2)	range (FO PET)	sum variance (GLCM PET)	difference variance (GLCM P4)

P1/2/3/4/5, dynamic phase 1/2/3/4/5; FO, first order; GLCM, gray level cooccurrence matrix; RLM, run length matrix; SZM, size zone matrix; NGLDM, neighborhood gray level dependence matrix; NGTDM, neighborhood gray tone difference

matrix; glnNorm, gray level nonuniformity normalized; srlgle, short run low gray level emphasis; lzhgle, large zone high gray level emphasis; lglze, low gray level large zone emphasis; coeffVar, coefficient of variation; hdhge, high dependence high gray level emphasis; hglze, high gray level large zone emphasis; dcv, dependence count variance; ldlge, low dependence low gray level emphasis; interQuartRange, interquartile range; szlgle, small zone high gray level emphasis; secondInfCorr, second information measure of correlation; lzlgle, large zone low gray level emphasis.

Table S11. Subtype (luminals vs. others).

Images	1st	2nd	3rd	4th	5th	6th
ADC	minimum (FO)	firstInfCorr (GLCM)	srlgle (RLM)	auto correlation (GLCM)	kurtosis (FO)	busyness (NGTDM)
T2	hdhge (NGLDM)	lglre (RLM)	coeffVar (FO)	correlation (GLCM)	sum entropy (GLCM)	busyness (NGTDM)
DCE 1	energy (NGLDM)	90th percentile (FO)	run length variance (RLM)	lzhgle (SZM)	inverse variance (GLCM)	secondInfCorr (GLCM)
DCE 2	entropy (FO)	lglze (SZM)	ldlge (NGLDM)	gray level variance (RLM)	sum variance (GLCM)	median (FO)
DCE 3	srlgle (RLM)	coeffDisp (FO)	sum variance (GLCM)	entropy (FO)	median (FO)	szlgle (SZM)
DCE 4	contrast (NGTDM)	median (FO)	entropy (FO)	sze (SZM)	firstInfCorr (GLCM)	gray level variance (SZM)
DCE 5	minimum (FO)	cluster prominence (GLCM)	firstInfCorr (GLCM)	energy (NGLDM)	range (FO)	coeffDisp (FO)
All DCE	minimum (FO P1)	median (FO P2)	coeffDisp (FO P1)	sze (SZM P5)	contrast (NGTDM P4)	coeffDisp (FO P5)
All MR	hdhge (NGLDM T2)	coeffVar (FO T2)	sze (SZM P5)	contrast (NGTDM P4)	median (FO P2)	lglre (RLM T2)
PET	inverse variance (GLCM)	sze (SZM)	difference entropy (GLCM)	secondInfCorr (GLCM)	dcnNorm (NGLDM)	sum entropy (GLCM)
All MR and PET	dcnNorm (NGLDM PET)	sze (SZM PET)	secondInfCorr (GLCM PET)	hdhge (NGLDM T2)	sze (SZM P5)	inverse variance (GLCM PET)

P1/2/3/4/5, dynamic phase 1/2/3/4/5; FO, first order; GLCM, gray level cooccurrence matrix; RLM, run length matrix; SZM, size zone matrix; NGLDM, neighborhood gray level dependence matrix; NGTDM, neighborhood gray tone difference matrix; firstInfCorr, first information measure of correlation; srlgle, short run low gray level emphasis; hdhge, high dependence high gray level emphasis; lglre, low gray level run emphasis; coeffVar, coefficient of variation; lzhgle, large zone high level emphasis; secondInfCorr, second information measure of correlation; lglze, low gray level zone emphasis; ldlge, low dependence low gray level emphasis; coeffDisp, coefficient of dispersion; szlgle, small zone low gray level emphasis; sze, small zone emphasis; dcnNorm, dependence count non-uniformity normalized.

Table S12. ER Status (negative vs. positive).

Images	1st	2nd	3rd	4th	5th	6th
ADC	secondInfCorr (GLCM)	10th percentile (FO)	root mean square (FO)	minimum (FO)	dcnNorm (NGLDM)	difference variance (GLCM)
T2	coeffVar (FO)	hdhge (NGLDM)	lglre (RLM)	gray level variance (SZM)	secondInfCorr (GLCM)	zln (SZM)
DCE 1	energy (FO)	median (FO)	complexity (NGTDM)	difference variance (GLCM)	kurtosis (FO)	lzlgle (SZM)
DCE 2	entropy (FO)	energy (FO)	coeffDisp (FO)	cluster shade (GLCM)	dcv (NGLDM)	zln (SZM)
DCE 3	entropy (FO)	zln (SZM)	dcv (NGLDM)	cluster prominence (GLCM)	energy (FO)	coeffDisp (FO)
DCE 4	contrast (NGTDM)	interQuartRange (FO)	90th percentile (FO)	dcv (NGLDM)	short run emphasis (RLM)	zln (SZM)
DCE 5	cluster shade (GLCM)	zln (SZM)	-	-	-	-
All DCE	minimum (FO P2)	minimum (FO P1)	cluster shade (GLCM P4)	difference variance (GLCM P1)	hdhge (NGLDM P1)	cluster shade (GLCM P3)
All MR	minimum (FO P2)	minimum (FO P1)	cluster shade (GLCM P4)	joint maximum (GLCM T2)	minimum (FO ADC)	gray level variance (SZM T2)
PET	inverse variance (GLCM)	difference variance (GLCM)	secondInfCorr (GLCM)	run length variance (RLM)	variance (FO)	lzhgle (SZM)
All MR and PET	minimum (FO P1)	median (FO P2)	coeffDisp (FO P1)	size zone emphasis (SZM P5)	contrast (NGTDM P4)	coeffDisp (FO P5)

P1/2/3/4/5, dynamic phase 1/2/3/4/5; FO, first order; GLCM, gray level cooccurrence matrix; RLM, run length matrix; SZM, size zone matrix; NGLDM, neighborhood gray level dependence matrix; NGTDM, neighborhood gray tone difference matrix; secondInfCorr, second information measure of correlation; dcnNorm, dependence count non-uniformity normalized; coeffVar, coefficient of variation; hdhge, high dependence high gray level emphasis; lglre, low gra y level run emphasis; zln, zone size non-uniformity; lzlgle, large zone low gray level emphasis; coeffDisp, coefficient of dispersion; dcv, dependence count variance; interQuartRange, interquartile range; lzhgle, large zone high gray level emphasis.

Table S13. PR status (negative vs. positive).

Images	1st	2nd	3rd	4th	5th	6th
ADC	10th percentile (FO)	90th percentile (FO)	lzhgle (SZM)	difference variance (GLCM)	-	-
T2	zln (SZM)	minimum (FO)	inverse variance (GLCM)	correlation (GLCM)	hgce (NGLDM)	hdhge (NGLDM)
DCE 1	lzlgle (SZM)	gray level variance (SZM)	secondInfCorr (GLCM)	kurtosis (FO)	entropy (FO)	minimum (FO)
DCE 2	minimum (FO)	zln (SZM)	10th percentile (FO)	-	-	-
DCE 3	lgce (NGLDM)	coeffDisp (FO)	10th percentile (FO)	gln (RLM)	entropy (FO)	variance (FO)
DCE 4	coeffDisp (FO)	hdhge (NGLDM)	complexity (NGTDM)	lgce (NGLDM)	contrast (NGTDM)	firstInfCorr (GLCM)
DCE 5	hdlge (NGLDM)	zln (SZM)	hdhge (NGLDM)	texture strength (NGTDM)	difference variance (GLCM)	interQuartRange (FO)
All DCE	hdhge (NGLDM P1)	minimum (FO P1)	hdlge (NGLDM P4)	gray level variance (SZM P1)	minimum (FO P2)	complexity (NGTDM P4)
All MR	10th percentile (FO ADC)	minimum (FO T2)	minimum (FO P2)	gray level variance (SZM P1)	90th percentile (FO ADC)	hdhge (NGLDM P1)
PET	zln (SZM)	sum entropy (GLCM)	szhgle (SZM)	lzhgle (SZM)	secondInfCorr (GLCM)	10th percentile (FO)
All MR and PET	zln (SZM PET)	mean (FO ADC)	minimum (FO T2)	10th percentile (FO P3)	gray level variance (SZM P1)	hdhge (NGLDM P4)

P1/2/3/4/5, dynamic phase 1/2/3/4/5; FO, first order; GLCM, gray level cooccurrence matrix; RLM, run length matrix; SZM, size zone matrix; NGLDM, neighborhood gray level dependence matrix; NGTDM, neighborhood gray tone difference matrix; lzhgle, large zone high gray level emphasis; zln, size zone non-uniformity; hgce, high gray level count emphasis, hdhge, high dependence high gray level emphasis; lzlgle, large zone low gray level emphasis; secondInfCorr, second information measure of correlation; lgce, low gray level count emphasis; coeffDisp, coefficient of dispersion; gln, gray level non-uniformity; lgce, low gray level count emphasis; firstInfoCorr, first information measure of correlation; hdlge, high dependence low gray level emphasis; interQuartRange, interquartile range; szhgle, small zone high gray level emphasis.

Table S14. HER2 status (negative vs. positive).

Images	1st	2nd	3rd	4th	5th	6th
ADC	glnNorm (RLM)	gray level variance (NGLDM)	zone emphasis (SZM)	szhgle (SZM)	cluster prominence (GLCM)	srlgle (RLM)
T2	szlgle (SZM)	complexity (NGTDM)	hdlge (NGLDM)	srlgle (RLM)	interQuartRange (FO)	joint maximum (GLCM)
DCE 1	entropy (NGLDM)	mean (FO)	energy (FO)	difference variance (GLCM)	texture strength (NGTDM)	complexity (NGTDM)
DCE 2	contrast (NGTDM)	short run emphasis (RLM)	texture strength (NGTDM)	gray level variance (SZM)	complexity (NGTDM)	inverse variance (GLCM)
DCE 3	contrast (NGTDM)	short run emphasis (RLM)	firstInfCorr (GLCM)	texture strength (NGTDM)	secondInfCorr (GLCM)	hdhge (NGLDM)
DCE 4	zln (SZM)	texture strength (NGTDM)	-	-	-	-
DCE 5	run emphasis (RLM)	gray level variance (SZM)	firstInfCorr (GLCM)	sum entropy (GLCM)	energy (GLCM)	szlgle (SZM)
All DCE	contrast (NGTDM P1)	texture strength (NGTDM P1)	minimum (FO P1)	szlgle (SZM P5)	mean (FO P1)	szhgle (SZM P4)
All MR	firstInfCorr (GLCM P1)	contrast (NGTDM P1)	range (FO T2)	szhgle (SZM P2)	szlgle (SZM P5)	90th percentile (FO P2)
PET	skewness (FO)	variance (FO)	hglze (SZM)	srlgle (RLM)	rlnNorm (RLM)	energy (GLCM)
All MR and PET	firstInfCorr (GLCM P1)	variance (FO)	energy (GLCM P1)	invDiffMomNorm (GLCM P4)	range (FO T2)	srlgle (RLM PET)

P1/2/3/4/5, dynamic phase 1/2/3/4/5; FO, first order; GLCM, gray level cooccurrence matrix; RLM, run length matrix; SZM, size zone matrix; NGLDM, neighborhood gray level dependence matrix; NGTDM, neighborhood gray tone difference matrix; glnNorm, gray level non-uniformity normalized; szhgle, small soze high gray level emphasis; srlgle, short run low gray level emphasis; szlgle, small zone low gray level emphasis; hdlge, high dependence low gray level emphasis; interQuartRange, interquartile range; firstInfCorr, first information measure of correlation; secondInfoCorr, second information measure of correlation; zln, size zone non-uniformity; hglze, high gray level zone emphasis; rlnNorm, run length non-uniformity normalized; invDiffMomNorm, inverse difference moment normalized.

Table S15. Proliferation (high vs. low).

Images	1st	2nd	3rd	4th	5th	6th
ADC	entropy (FO)	correlation (GLCM)	run length variance (RLM)	sum variance (GLCM)	difference variance (GLCM)	run emphasis (RLM)
T2	total energy (FO)	complexity (NGTDM)	szlgle (SZM)	variance (FO)	size zone emphasis (SZM)	gray level variance (RLM)
DCE 1	haralick Correlation (GLCM)	glnNorm (RLM)	sum entropy (GLCM)	correlation (GLCM)	maximum (FO)	invDiffNorm (GLCM)
DCE 2	correlation (GLCM)	firstInfCorr (GLCM)	hdlge (NGLDM)	90th percentile (FO)	entropy (FO)	cluster prominence (GLCM)
DCE 3	coeffVar (FO)	correlation (GLCM)	gray level variance (SZM)	hdhge (NGLDM)	glnNorm (SZM)	firstInfCorr (GLCM)
DCE 4	run emphasis (RLM)	entropy (NGLDM)	invDiffMom (GLCM)	invDiffMomNorm (GLCM)	gray level variance (SZM)	correlation (GLCM)
DCE 5	glnNorm (SZM)	entropy (FO)	median (FO)	correlation (GLCM)	gray level variance (SZM)	sum variance (GLCM)
All DCE	inverse variance (GLCM P2)	gray level variance (SZM P3)	entropy (FO P5)	joint variance (GLCM P4)	minimum (FO P2)	cluster shade (GLCM P4)
All MR	gray level variance (SZM P3)	minimum (FO P2)	contrast (GLCM P4)	minimum (FO T2)	firstInfCorr (GLCM P1)	secondInfCorr (GLCM ADC)
PET	range (FO)	zln (SZM)	energy (NGLDM)	secondInfCorr (GLCM)	joint entropy (GLCM)	entropy (FO)
All MR and PET	gray level variance (SZM P3)	inverse variance (GLCM P4)	minimum (FO P2)	difference variance (GLCM P1)	szlgle (SZM T2)	hdhge (NGLDM PET)

P1/2/3/4/5, dynamic phase 1/2/3/4/5; FO, first order; GLCM, gray level cooccurrence matrix; RLM, run length matrix; SZM, size zone matrix; NGLDM, neighborhood gray level dependence matrix; NGTDM, neighborhood gray tone difference matrix; szlgle, small zone low gray level emphasis; glnNorm, gray level non-uniformity normalized; invDiffNorm, inverse difference normalized; firstInfoCorr, first information measure of correlation; hdlge, high dependence low gray level emphasis; coeffVar, coefficient of variance; hdhge, high dependence high gray level emphasis; invDiffMomNorm, inverse difference moment normalized; secondInfCorr, second information measure of correlation; zln, size zone non-uniformity.

Table S16. Grade (grade 1 and 2 vs. Grade 3).

Images	1st	2nd	3rd	4th	5th	6th
ADC	firstInfCorr (GLCM)	maximum (FO)	secondInfCorr (GLCM)	-	-	-
T2	szlgle (SZM)	texture strength (NGTDM)	10th percentile (FO)	gray level variance (SZM)	lgce (NGLDM)	range (FO)
DCE 1	texture strength (NGTDM)	rlv (RLM)	energy (FO)	-	-	-
DCE 2	minimum (FO)	coarseness (NGTDM)	meanAbsDev (FO)	dcv (NGLDM)	root mean square (FO)	hdlge (NGLDM)
DCE 3	hdlge (NGLDM)	texture strength (NGTDM)	minimum (FO)	interQuartRange (FO)	dcv (NGLDM)	Haralick correlation (GLCM)
DCE 4	root mean square (FO)	coarseness (NGTDM)	dcv (NGLDM)	szlgle (SZM)	minimum (FO)	Haralick correlation (GLCM)
DCE 5	coarseness (NGTDM)	mean (FO)	difference variance (GLCM)	contrast (GLCM)	-	-
All DCE	root mean square (FO P3)	minimum (FO P2)	minimum (FO P1)	run length variance (RLM P1)	coarseness (NGTDM P3)	Haralick correlation (GLCM P3)
All MR	minimum (FO P2)	minimum (FO P1)	root mean square (FO P3)	10th percentile (FO T2)	szlgle (SZM T2)	texture strength (NGTDM T2)
PET	zln (SZM)	entropy (FO)	glnNorm (SZM)	hdhge (NGLDM)	10th percentile (FO)	Haralick correlation (GLCM)
All MR and PET	glnNorm (SZM PET)	minimum (FO P1)	10th percentile (FO T2)	zln (SZM PET)	szlgle (SZM T2)	minimum (FO P3)

P1/2/3/4/5, dynamic phase 1/2/3/4/5; FO, first order; GLCM, gray level cooccurrence matrix; RLM, run length matrix; SZM, size zone matrix; NGLDM, neighborhood gray level dependence matrix; NGTDM, neighborhood gray tone difference matrix; firstInfCorr, first information measure of correlation; secondInfCorr, second information measure of correlation; szlgle, small zone low gray level emphasis; lgce, low gray level count emphasis; rlv, run length variance; meanAbsDev, mean absolute deviation; dcv, dependence count variance; hdlge, high dependence low gray level emphasis; interQuartRange, interquartile range; zln, size zone non-uniformity; glnNorm, gray level non-uniformity normalized; hdhge, high dependence high gray level emphasis.

Table S17. Nodal Status (0 vs. 1,2,3).

Images	1st	2nd	3rd	4th	5th	6th
ADC	szlgle (SZM)	interQuartRange (FO)	difference entropy (GLCM)	skewness (FO)	energy (GLCM)	hde (NGLDM)
T2	texture strength (NGTDM)	hglze (SZM)	median (FO)	skewness (FO)	secondInfCorr (GLCM)	busyness (NGTDM)
DCE 1	texture strength (NGTDM)	correlation (GLCM)	dcv (NGLDM)	difference variance (GLCM)	minimum (FO)	lzlgle (SZM)
DCE 2	difference variance (GLCM)	ldlge (NGLDM)	gray level variance (RLM)	texture strength (NGTDM)	busyness (NGTDM)	10th percentile (FO)
DCE 3	difference variance (GLCM)	ldlge (NGLDM)	gray level variance (RLM)	coarseness (NGTDM)	-	-
DCE 4	difference variance (GLCM)	coarseness (NGTDM)	ldlge (NGLDM)	minimum (FO)	gray level variance (SZM)	joint variance (GLCM)
DCE 5	difference variance (GLCM)	coarseness (NGTDM)	szlgle (SZM)	energy (NGLDM)	-	-
All DCE	ldlge (NGLDM P2)	difference variance (GLCM P3)	gray level variance (RLM P3)	coarseness (NGTDM P3)	difference variance (GLCM P2)	10th percentile (FO P1)
All MR	szlgle (SZM ADC)	interQuartRange (FO ADC)	energy (GLCM ADC)	difference entropy (GLCM ADC)	difference variance (GLCM P2)	gray level variance (SZM P3)
PET	firstInfCorr (GLCM)	correlation (GLCM)	energy (GLCM)	energy (FO)	median (FO)	joint entropy (GLCM)
All MR and PET	interQuartRange (FO ADC)	szlgle (SZM ADC)	difference variance (GLCM P2)	energy (GLCM ADC)	difference entropy (GLCM ADC)	dcnNorm (NGLDM T2)

P1/2/3/4/5, dynamic phase 1/2/3/4/5; FO, first order; GLCM, gray level cooccurrence matrix; RLM, run length matrix; SZM, size zone matrix; NGLDM, neighborhood gray level dependence matrix; NGTDM, neighborhood gray tone difference matrix; szlgle, small zone low gray level emphasis; interQuartRange, interquartile range; hde, high dependence emphasis; hglze, high gray level zone emphasis; secondInfoCorr, second information measure of correlation; dcv, dependence count variance; lzlgle, large zone low gray level emphasis; ldlge, low dependence low gray level emphasis; firstInfCorr, first information measure of correlation; dcnNorm, dependence count non-uniformity normalized.

Table S18. Metastases (0 vs. 1).

Images	1st	2nd	3rd	4th	5th	6th
ADC	joint maximum (GLCM)	coarseness (NGTDM)	entropy (NGLDM)	gray level variance (NGLDM)	lzlgle (SZM)	glnNorm (SZM)
T2	texture strength (NGTDM)	zone emphasis (SZM)	hdhge (NGLDM)	maximum (FO)	gray level variance (SZM)	cluster shade (GLCM)
DCE 1	texture strength (NGTDM)	dcnNorm (NGLDM)	szlgle (SZM)	coeffDisp (FO)	run length variance (RLM)	kurtosis (FO)
DCE 2	gln (RLM)	energy (FO)	firstInfCorr (GLCM)	coarseness (NGTDM)	complexity (NGTDM)	glnNorm (RLM)
DCE 3	coarseness (NGTDM)	joint maximum (GLCM)	gln (RLM)	energy (FO)	energy (NGLDM)	szlgle (SZM)
DCE 4	busyness (NGTDM)	coarseness (NGTDM)	sum variance (GLCM)	complexity (NGTDM)	energy (FO)	energy (NGLDM)
DCE 5	complexity (NGTDM)	coarseness (NGTDM)	joint maximum (GLCM)	firstInfCorr (GLCM)	gln (RLM)	energy (FO)
All DCE	dcnNorm (NGLDM P1)	firstInfCorr (GLCM P4)	complexity (NGTDM P5)	minimum (FO P1)	texture strength (NGTDM P1)	10th percentile (FO P4)
All MR	texture strength (NGTDM ADC)	joint entropy (GLCM ADC)	minimum (FO P2)	variance (FO ADC)	cluster prominence (GLCM ADC)	szlgle (SZM P2)
PET	energy (NGLDM)	hgce (NGLDM)	minimum (FO)	lzlgle (SZM)	gln (SZM)	root mean square (FO)
All MR and PET	texture strength (NGTDM ADC)	joint entropy (GLCM ADC)	joint entropy (GLCM PET)	inverse variance (GLCM P2)	secondInfCorr (GLCM ADC)	minimum (FO P1)

P1/2/3/4/5, dynamic phase 1/2/3/4/5; FO, first order; GLCM, gray level cooccurrence matrix; RLM, run length matrix; SZM, size zone matrix; NGLDM, neighborhood gray level dependence matrix; NGTDM, neighborhood gray tone difference matrix; lzlgle, large zone low gray level emphasis; glnNorm, gray level non-uniformity normalized; hdhge, high dependence high gray level emphasis; dcnNorm, dependence count non-uniformity normalized; szlgle, small zone low gray level emphasis; coeffDisp, coefficient of dispersion; gln, gray level non-uniformity; firstInfCorr, first information measure of correlation, szlgle, small zone low gray level emphasis; hgce, high gray level count emphasis; secondInfCorr, second information measure of correlation.
